# Altered cerebral blood flow patterns in ankylosing spondylitis: A three-dimensional pseudo-continuous arterial spin labeling study

**DOI:** 10.3389/fnins.2022.961489

**Published:** 2022-08-03

**Authors:** Jin Fang, Kelei Hua, Feng Chen, Zhifang Wan, Yi Yin, Ping Liu, Tianyue Wang, Guihua Jiang

**Affiliations:** ^1^The Second School of Clinical Medicine, Southern Medical University, Guangzhou, China; ^2^Department of Medical Imaging, Guangdong Second Provincial General Hospital, Guangzhou, China; ^3^Department of Medical Image Center, Yuebei People’s Hospital, Shaoguan, China

**Keywords:** ankylosing spondylitis (AS), cerebral blood flow (CBF), arterial spin labeling (ASL), brain perfusion, magnetic resonance imaging (MRI)

## Abstract

**Objective:**

This study aimed to detect the cerebral blood flow (CBF) values changes in patients with ankylosing spondylitis (AS) and to evaluate the correlation between the CBF values and the specific clinical characteristics.

**Materials and methods:**

Forty-eight patients with AS (43 male and 5 female) and 42 healthy controls (HCs) (38 male and 4 female) were recruited. Three-dimensional pseudo-continuous arterial spin labeling (3D-pCASL) was performed on a 3.0T magnetic resonance imaging (MRI). CBF values were obtained on the Philips post-processing workstation based on arterial spin labeling (ASL) data. The two-sample *t*-test was used to compare CBF differences. The correlation between CBF values and specific clinical characteristics of AS was evaluated.

**Results:**

The AS group showed increased CBF values in the right precentral gyrus, the left inferior frontal gyrus, and the left temporal pole compared with HCs the AS group also showed decreased CBF values in the left precuneus and the left superior occipital gyrus compared with HCs. There were no significant correlations between the CBF values and the clinical characteristics including total back pain (TBP), erythrocyte sedimentation rate (ESR), and C-reactive protein (CRP).

**Conclusion:**

Patients with AS displayed CBF changes compared with HCs using 3D-PCASL. These results may enhance our understanding of the neural substrates of AS and provide evidence of AS-related neurological impairment.

## Introduction

Ankylosing spondylitis (AS) is the major subtype of an inter-related group of rheumatic diseases named spondyloarthritis (SpA) that affects the axial skeleton and other joints and organs (Braun and Sieper, 2007). The global incidence of AS is about 0.9% and it mainly occurs in young patients with a male-to-female ratio of roughly 2 to 1 (Feldtkeller et al., 2003). The main clinical manifestation of AS is lower back pain in the early stage and motion limitation due to stiffness of the spine in the late stage. In terms of etiology, AS may be the result of the dual effects of environmental factors and genetic inheritance, the most important of which is HLA-B27 (Bowness, 2015), but the exact pathogenesis is still unknown.

Increasingly studies have found brain structural and functional changes in AS (Cidem et al., 2014; Hemington et al., 2016). From the brain structural perspective, the gray matter volume in the left putamen in patients with AS increased significantly and was positively correlated with the duration of AS and total back pain (TBP) scores, whereas it was not correlated with bath ankylosing spondylitis disease activity index (BASDAI) scores, C-reactive protein (CRP), or erythrocyte sedimentation rate (ESR) (Hua et al., 2020). From the brain functional perspective, A whole-network analysis revealed that AS patients exhibited less anticorrelated functional connectivity (FC) between the salience network and the default mode network, suggesting that cross-network FC is a metric of functional brain abnormality in AS (Hemington et al., 2016). Another FC and low-frequency fluctuations (ALFF) study found that the FCs of the left middle temporal gyrus and left precuneus of AS patients were closely related to BASDAI scores, ESR, and CRP. Additionally, the ALFF values of multiple brain areas were significantly changed in AS patients (Li et al., 2017).

Previous studies have also found a relationship between pain disorders and cerebral perfusion using the method of arterial spin labeling (ASL). In chronic knee pain, significant hypo-perfusion was found in the anterior medial prefrontal cortex, the angular gyrus, and the ventral anterior insular cortex, while hyper-perfusion was found in posterior default mode, thalamus, and sensory regions (Iwabuchi et al., 2020). Perfusion changes in different brain regions in patients with chronic pain provided valuable insights into the pathogenesis of AS since the brain perfusion difference can reflect the level of neural activity.

Therefore, in the present study, we used the three-dimensional pseudo-continuous ASL (3D-pCASL) method to detect the brain cerebral blood flow (CBF) differences between patients with AS and healthy controls (HCs). We also evaluated the correlation between CBF values and the clinical characteristics of AS. We hope our results will help to explore the underlying pathophysiology of AS.

## Materials and methods

### Subjects

A total of 52 patients with AS were recruited from the Guangdong Second Provincial General Hospital. 44 sex- and age-matched HCs were recruited through local advertisements. All participants were right-handed. The inclusion criteria for AS patients were as follows: (1) diagnosis of active AS according to the revised New York criteria; (2) non-steroidal anti-inflammatory drugs (NSAIDs) were only taken in stable doses for pain; (3) did not take biological agents during the study or at any other time; (4) no comorbidities, such as anxiety, presence of fibromyalgia, and depression and so on; and (5) the average TBP score of the week before the report was ≥3 (out of 10, 0 = no pain, 10 = the most severe pain imaginable). The inclusion criteria for HCs were as follows: (1) 16–55 years old; (2) no prior diagnosis of neurological disease or mental illness; (3) no malignant disease in the past 2 years; (4) no pregnancy or no breastfeeding; and (5) no other MRI contraindications.

### Magnetic resonance imaging data acquisition

The image data were performed on a 3.0T MRI scanner (Ingenia; Philips, Best, Netherlands). In the resting-state perfusion imaging, the pCASL sequence was used for three-dimensional (3D) fast spin-echo acquisition and background suppression. The acquisition parameters were the following: echo time (TE) = 33 ms, repetition time (TR) = 4,155 ms, field of view (FOV) = 240 mm^2^ × 240 mm^2^, post-labeling delay (PLD) = 2,000 ms, in-plane voxel size = 3.75 × 3.75 × 6.00, in-plane matrix = 64 × 59, slice thickness/gap = 6.0/0 mm, 20 axial slices covering the whole brain, NSA = 1, and acquisition time = 4 min 51 s. In addition, a 3D T1-weighted brain volume imaging sequence covering the whole brain was used for structural data acquisition with: TR/TE = 7.8/3.6 ms, slice thickness/gap = 1.0/0 mm, flip angle = 8, NSA = 1, matrix = 256 × 256, FOV = 240 mm × 240 mm, 185 sagittal slices, and acquisition time = 5 min 56 s. Routine MRI images were evaluated by two experienced neuroradiologists to confirm that there were no brain structural abnormalities.

### Cerebral blood flow processing

The pCASL images were analyzed on a Philips post-processing workstation. Quantification of CBF was calculated with the equation:


(1) $$CBF=\frac{6000\cdot \lambda \cdot \left(SI_{control}-SI_{label}\right)\cdot e^{PLD/T_{1,blood}}}{2\cdot a\cdot T_{1,blood}\cdot SI_{PD}\cdot (1-e^{-\tau /T_{1,blood}}}$$

where T1 of blood (T_1, blood_) was assumed to be 1,650 ms at 3.0T, labeling efficiency (α) 0.85, partition coefficient (λ) 0.9, PLD 2,000 ms, and labeling duration (τ) 1,800 ms. SI_PD_ is the signal intensity of a proton density weighted image, SI_control_ is the time-averaged signal intensities in the control label images, and SI_label_ is the time-averaged signal intensities in the label images. The CBF maps were normalized to the standard space of the Montreal Neurological Institute (MNI) using the Statistical Parametric Mapping (SPM12)^1^ software: (1) Co-registration of the individual CBF brain map and the individual 3D T1-weighted structural image to obtain the individual T1’ brain map. (2) In the standard space, the T1 brain maps of all individuals are nonlinearly normalized to T1 templates. (3) All the CBF images are normalized to MNI space by using the normalization parameters estimated in step 2, and resampled to the voxel size of 2 mm × 2 mm × 2 mm. (4) The CBF value of each voxel was transformed by *z* transformation: (single voxel CBF – mean CBF of the whole brain)/standard deviation of the whole brain CBF. (5) The CBF maps were smoothed using a Gaussian smoothing kernel with a full width at half maximum of 6 mm.

### Statistical analysis

SPSS for Windows version 22.0 (SPSS Inc., Chicago, IL, United States) was used for statistical analysis. Independent-sample *t*-tests were used to compare age, TBP, ESR, and CRP. Gender differences between the two groups were compared using the 𝒳^2^ test. All tests were the two-tailed test, *p* < 0.05 was considered to be statistically significant.

In order to compare the CBF maps, the CBF maps between the AS group and the HCs group, a voxel-based comparison was made, using the *t*-test of the two samples, and the individual’s age and sex were used as nuisance covariates. The voxel-level correction of family-wise error (FWE) for multiple comparisons was used in all group comparisons.

Save the clusters that show significant group differences on the CBF maps between the two groups as a binary mask to extract CBF values. We then calculated the partial correlation analysis between these CBF values and TBP, ESR, and CRP. We used gender and age as covariates. Multiple comparisons were performed using the Bonferroni correction.

## Results

### Demographic information

Demographic information and clinical characteristics of all recruited participants are shown in Table 1. Two HCs and Four patients with AS were excluded from further analyses because of image artifacts. Finally, 48 AS patients and 42 HCs were included. There were no significant differences in age and sex between the two groups.

**TABLE 1:** Demographic and clinical data comparisons.

Characteristics	AS	HC	Statistics	*P*-value
Case	48	42	N/A	N/A
Gender (M/F)	43/5	38/4	χ^2^ = 0.020*^a^*	0.888
Age (years old)	25.9 ± 6.4	28.1 ± 3.7	*T* = 8.460*^b^*	0.052
TBP	6.0 ± 1.4	N/A	N/A	N/A
ESR	14.3 ± 7.7	N/A	N/A	N/A
CRP	11.7 ± 9.1	N/A	N/A	N/A

Means and standard deviations (SD) are listed in the table.

AS, ankylosing spondylitis; HC, healthy control; M, male; F, female; TBP, total back pain; ESR, erythrocyte sedimentation rate; CRP, C-reactive protein; N/A, not applicable.

^*a*^χ^2^-test.

^*b*^Two-sample *t*-test.

### Differences in cerebral blood flow values between two groups

**FIGURE 1:**
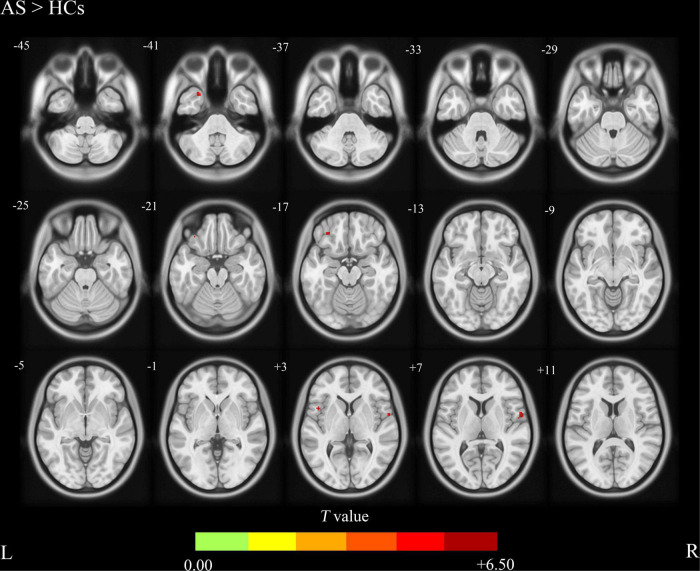
Ankylosing spondylitis patients showed significantly increased CBF clusters than HCs (voxel-level correction of FWE). AS, ankylosing spondylitis; HCs, healthy controls; CBF, cerebral blood flow; L (R), left (right) hemisphere; The color bar indicates the T value from the two-sample *t*-test.

**FIGURE 2:**
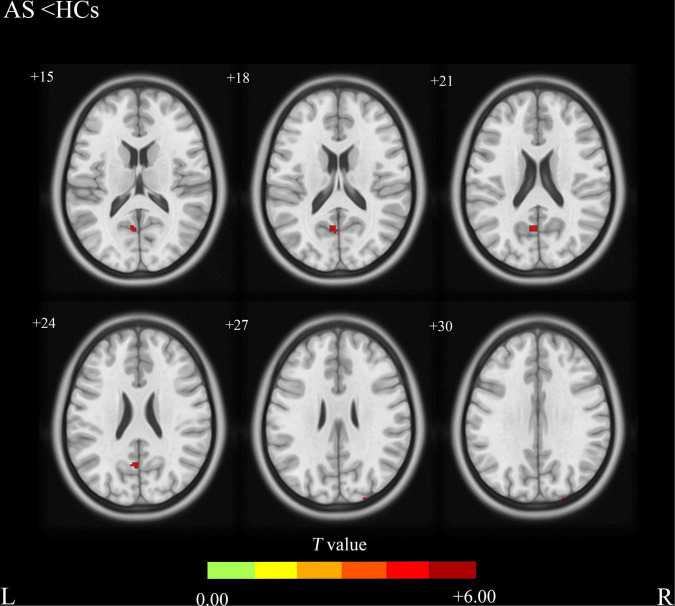
Ankylosing spondylitis patients showed significantly decreased CBF clusters than HCs (voxel-level correction of FWE). AS, ankylosing spondylitis; HCs, healthy controls; CBF, cerebral blood flow; L (R), left (right) hemisphere; The color bar indicates the T value from the two-sample *t*-test.

Compared with the HCs group, the AS group showed increased CBF values (Table 2 and Figure 1) in the right precentral gyrus, the left inferior frontal gyrus, and the left Temporal Pole, and showed decreased CBF values in the left precuneus and the superior occipital gyrus (Table 2 and Figure 2).

**TABLE 2:** The areas of significantly different CBF values between the AS patients and the HCs (voxel-level correction of FWE).

Brain regions	Brodman area	Montreal neurological institute coordinates			Peak *t*-value	Cluster size (voxel numbers)
		X	Y	Z		
** CBF values: AS > HCs**						
R precentral gyrus	48	58	4	8	6.37	32
L inferior frontal gyrus	47	–32	38	–18	5.69	19
L inferior frontal gyrus	48	–46	12	4	5.61	9
L temporal pole	36	–28	14	–40	5.51	13
** CBF values: AS < HCs**						
L precuneus	23	–6	–62	18	5.81	59
R superior occipital gyrus	18	24	–94	32	5.44	8

CBF, cerebral blood flow; AS, ankylosing spondylitis; HC, healthy control; FWE, family-wise error; L (R), left (right) hemisphere.

### Correlation analysis

Partial correlation analysis showed that there were no significant correlations between the right precentral gyrus, the left inferior frontal gyrus, the left Temporal Pole, the left precuneus, the superior occipital gyrus, and the TBP, ESR, and CRP (Table 3).

**TABLE 3:** Analysis of partial correlation between abnormal CBF values and clinical information.

		TBP	ESR	CRP
CBF values of the R precentral gyrus	*r*	0.161	–0.022	–0.016
	*p*	0.285	0.887	0.918
CBF values of the L inferior frontal gyrus	*r*	–0.034	0.201	0.281
	*p*	0.822	0.180	0.059
CBF values of the L inferior frontal gyrus	*r*	0.004	0.262	0.088
	*p*	0.981	0.079	0.559
CBF values of the L temporal pole	*r*	0.055	0.127	0.222
	*p*	0.717	0.399	0.138
CBF values of the L precuneus	*r*	–0.033	–0.223	–0.038
	*p*	0.825	0.137	0.800
CBF values of the R superior occipital gyrus	*r*	–0.259	0.121	–0.093
	*p*	0.082	0.422	0.537

CBF, cerebral blood flow; TBP, total back pain; ESR, erythrocyte sedimentation rate; CRP, C-reactive protein; L (R), left (right) hemisphere.

## Discussion

In this study, we explored the CBF changes in AS patients and the relationship between the altered CBF and the clinical characteristics including TBP, ESR, and CRP. We found increased CBF values in the right precentral gyrus, the left inferior frontal gyrus and the left temporal pole in AS patients compared with the HCs. In addition, our findings showed that AS patients exhibited decreased CBF values in the left precuneus and the superior occipital gyrus compared with HCs. There were no significant correlations between CBF values changes in the right precentral gyrus, the left inferior frontal gyrus, the left Temporal Pole, the left precuneus, the superior occipital gyrus, and the TBP, ESR, and CRP.

To the best of our knowledge, this study is the first to investigate the CBF changes in AS using ASL. We found that AS patients exhibited significantly increased CBF values in the right precentral gyrus, the left inferior frontal gyrus, and the left temporal pole compared with the HCs. These altered CBF values in the brain regions described above may reflect the possible characteristics of neurological changes of AS. The precentral gyrus, also known as the primary motor cortex, is a very important structure involved in executing voluntary motor movements (Banker and Tadi, 2021). A previous functional MRI (fMRI) study found significantly lower ALFF in the right precentral gyrus (Li et al., 2017) and we found increased CBF in the same brain region. Lesions of the precentral gyrus can result in paralysis of the corresponding limbs or trunk (Pikula et al., 2011). The increased CBF values in the precentral gyrus may be a compensatory mechanism for voluntary motor movement limitation in AS patients. This compensatory mechanism has also been reported in other motor restriction disorders (Sabatini et al., 2000; Mallol et al., 2007). The inferior frontal gyrus is limited above by the inferior frontal sulcus and below by the external border of the hemisphere in the front, and by the Sylvian fissure behind (Wagner et al., 2013). The inferior frontal gyrus is responsible for the motor component of speech in the dominant hemisphere, which involves related functions of the lips, tongue, larynx, and pharynx coordinated to produce phonation (Hartwigsen et al., 2019). Interestingly, we found the CBF values increased in the left frontal gyrus because it seems that there is no relationship between the function of this brain area and AS. There is growing interest regarding the role of the inferior frontal gyrus during a particular form of executive control referred to as response inhibition (Paulus et al., 2002; Picton et al., 2007; Prodoehl et al., 2008; Hampshire et al., 2010). As pain is one of the main symptoms of AS patients, the voluntary motor movement pattern may be influenced by the region of the inferior frontal lobe. The temporal pole is a complex anatomical region with several distinct areas in the anterior part of the temporal lobe, each part of which has specific cytoarchitectural organization and connectivity patterns, and it has been associated with many different functions (Dupont, 2002; Herlin et al., 2021). The increased CBF values in the left temporal pole of AS patients may be associated with the socio-emotional function of the temporal pole, because chronic pain, activity limitation, and even kyphosis in the late stage of the disease may cause some psychological problems (Martindale et al., 2006; Brionez et al., 2009; Yurdakul et al., 2017).

In this study, we also found significantly decreased CBF values in the left precuneus and the superior occipital gyrus in AS patients compared with HCs. The precuneus is a brain region involved in a variety of complex functions and it is a functional core of the default-mode network (DMN) (Utevsky et al., 2014). Previous fMRI studies have proved that DMN is involved in the integration of autobiographical, self-monitoring, and social cognitive functions (Spreng et al., 2009). In addition, DMN may also participate in the central processing of fatigue or pain-related signals. Investigators have identified some brain alterations in DMN regions in migraine (Liu et al., 2015), fibromyalgia (Pujol et al., 2014), and knee osteoarthritis (Pujol et al., 2017). The decreased CBF values of the precuneus exhibited in our study may suggest the impairment of DMN which is common in chronic pain-related diseases and is consistent with the previous study (Amiri et al., 2021). The superior occipital gyrus is continuous along the superomedial margin of the hemisphere with the cuneus and is involved in many complex functions of the human body. The decreased CBF value in the superior occipital gyrus may reflect a neurological function decrease in this area, but the pathophysiology is still unclear and further studies are needed.

At last, we evaluated the correlation between the altered CBF values and clinical characteristics of AS including TBP, ESR, and CRP. It showed that there were no significant correlations between the CBF values and the TBP, ESR, and CRP. These results were in part consistent with previous studies (Li et al., 2017; Hua et al., 2020), indicating that those CBF value changes may represent a general basic brain functional transformation of AS.

There were several limitations in this study. First, in this cross-sectional study, we found the CBF values changed in several brain regions in AS patients, but the directionality of the relationship between AS and altered CBF values remains unclear, future longitudinal studies are needed to resolve this question. Second, because of the cross-sectional group data, we were unable to observe dynamic CBF values change over the developmental course of AS. Third, the sample of this study was small and the gender bias was large. Thus, the gender differences in brain CBF patterns may influence the results. Future studies should address these issues through longitudinal assessment of a large and gender-balanced sample of AS patients.

## Conclusion

Our preliminary study explored CBF values change in AS patients compared with HCs using 3D-PCASL. Some of the increased and decreased CBF values in different brain regions are consistent with previous fMRI studies. These results may enhance our understanding of the neural substrates of AS and provide evidence of AS-related neurological impairment. Hence, further investigation of the pathophysiology of the regions with altered CBF values is warranted.

## Data availability statement

The original contributions presented in this study are included in the article/supplementary material, further inquiries can be directed to the corresponding author/s.

## Ethics statement

The studies involving human participants were reviewed and approved by Ethics Committee of Guangdong Second Provincial General Hospital. Written informed consent to participate in this study was provided by the participants’ legal guardian/next of kin. Written informed consent was obtained from the individual(s), and minor(s)’ legal guardian/next of kin, for the publication of any potentially identifiable images or data included in this article.

## Author contributions

GJ designed the experiment. JF carried out the experiment and wrote the manuscript. JF, KH, and FC collected and sorted out the data. YY, ZW, PL, and TW helped with data management and processing. All authors contributed to the article and approved the submitted version.

## Footnotes

1
http://www.fil.ion.ucl.Ac.uk/spm
